# Revealing the Complete Bispecific Phosphatase Genes (DUSPs) across the Genome and Investigating the Expression Patterns of GH_A11G3500 Resistance against *Verticillium wilt*

**DOI:** 10.3390/ijms25084500

**Published:** 2024-04-19

**Authors:** Yahui Deng, Xiaojuan Deng, Jieyin Zhao, Shuo Ning, Aixing Gu, Quanjia Chen, Yanying Qu

**Affiliations:** College of Agronomy, Xinjiang Agricultural University, 311 Nongda East Road, Urumqi 830052, China; huiyad0330@163.com (Y.D.); dengxj007@163.com (X.D.); cottonzjy@126.com (J.Z.); sure.n@outlook.com (S.N.); gax@xjau.edu.cn (A.G.); chqjia@126.com (Q.C.)

**Keywords:** upland cotton, *Verticillium wilt*, bispecific protein phosphatase, phosphorylation, Mitogen-Activated Protein Kinase

## Abstract

DUSPs, a diverse group of protein phosphatases, play a pivotal role in orchestrating cellular growth and development through intricate signaling pathways. Notably, they actively participate in the MAPK pathway, which governs crucial aspects of plant physiology, including growth regulation, disease resistance, pest resistance, and stress response. DUSP is a key enzyme, and it is the enzyme that limits the rate of cell metabolism. At present, complete understanding of the DUSP gene family in cotton and its specific roles in resistance to *Verticillium wilt* (VW) remains elusive. To address this knowledge gap, we conducted a comprehensive identification and analysis of four key cotton species: *Gossypium arboreum*, *Gossypium barbadense*, *Gossypium hirsutum*, and *Gossypium raimondii*. The results revealed the identification of a total of 120 DUSP genes in the four cotton varieties, which were categorized into six subgroups and randomly distributed at both ends of 26 chromosomes, predominantly localized within the nucleus. Our analysis demonstrated that closely related DUSP genes exhibited similarities in terms of the conserved motif composition and gene structure. A promoter analysis performed on the GhDUSP gene promoter revealed the presence of several cis-acting elements, which are associated with abiotic and biotic stress responses, as well as hormone signaling. A tissue expression pattern analysis demonstrated significant variations in GhDUSP gene expression under different stress conditions, with roots exhibiting the highest levels, followed by stems and leaves. In terms of tissue-specific detection, petals, leaves, stems, stamens, and receptacles exhibited higher expression levels of the GhDUSP gene. The gene expression analysis results for GhDUSPs under stress suggest that DUSP genes may have a crucial role in the cotton response to stress in cotton. Through Virus-Induced Gene Silencing (VIGS) experiments, the silencing of the target gene significantly reduced the resistance efficiency of disease-resistant varieties against *Verticillium wilt* (VW). Consequently, we conclude that *GH_A11G3500*-mediated bispecific phosphorylated genes may serve as key regulators in the resistance of *G. hirsutum* to *Verticillium wilt* (VW). This study presents a comprehensive structure designed to provide an in-depth understanding of the potential biological functions of cotton, providing a strong foundation for further research into molecular breeding and resistance to plant pathogens.

## 1. Introduction

*Verticillium dahliae*, a soil pathogen that is spread through soil and has a significant effect on the yield of cotton (*G. hirsutum*), is responsible for causing the destructive plant disease known as *Verticillium wilt* [1]. The threat posed by this fungal infection necessitates the identification and characterization of novel genes implicated in resistance against *Verticillium dahliae*, which is crucial information for breeding resistant or tolerant cotton varieties [2].

The Mitogen-Activated Protein Kinase (MAPK) cascade, an essential cellular signal transduction module, consists of three key components: MAPKKK/MEKK, MAPKK/MKK/MEK, and MAPK [3,4]. Within this cascade, dual-specificity phosphatase (DUSP) functions as a protein phosphatase that specifically regulates MAPK activity through facilitating dephosphorylation processes [5,6]. DUSP plays a pivotal role in maintaining cellular homeostasis, immune response, and apoptosis. Its subfamilies share common features, including the N-terminal MAPK binding (MKB) domain, which serves as an essential component of the catalytic site and facilitates Thr and Tyr dephosphorylation of MAPK through interaction with the docking domain of MAPK [7,8,9]. Additionally, DUSP regulates MAPK signaling by competing with its substrates, as both the substrates of MAPK and DUSP interact with it via a shared docking domain [10]. Members of the DUSP family can be categorized into two groups: classical MAPK phosphatases and atypical DUSPs. Both play crucial roles in regulating tumor-associated MAP kinases in the context of the broader MAPK pathway [11,12,13].

The investigation and functional analysis of dual-specific protein phosphatases in plants is a burgeoning field, particularly with regard to their response to biological stress conditions [14]. These genetic factors are crucial in the regulation of plant growth and development, tissue specialization, organ formation, and various fundamental biological processes [15,16]. Concurrently, the research conducted on dual-specificity phosphatases (DUSPs) in the field of human diseases encompasses a broad spectrum of aspects, including cancer, inflammation, neurological disorders, and metabolic ailments [17]. For instance, elevated levels of *DUSP1* expression have been associated with inhibiting apoptosis in tumor cells and promoting the growth and metastasis of cancer cells [18,19,20]. Conversely, reduced levels of *DUSP4* (MKP-2) expression have been linked to suppressing cellular proliferation and facilitating cell apoptosis across various malignancies [21,22]. The abnormal expression patterns observed in *DUSP26* are correlated with Parkinson’s disease pathogenesis and neuronal impairment [23]. Moreover, it is plausible that *DUSP9* may be implicated in metabolic disorders, such as diabetes and obesity, due to its correlation with adipose tissue metabolism [24,25]. Subsequent investigations revealed that the upregulation of *DUSP16* in neoplastic cells conferred augmented resistance to apoptotic cell death following chemotherapy, whereas the downregulation of *DUSP16* enhanced the sensitivity of cancer cells toward treatment [26]. Mechanistically, *DUSP16* impedes JNK and p38 activation, thereby attenuating BAX accumulation within mitochondria and subsequently retarding apoptosis progression [27]. In mice, *DUSP1* (MKP-1) exerts a negative regulatory role in immune cells and is closely associated with the immune response and inflammation [28]. Simultaneously, *DUSP16* also participates in the regulation of nervous system development and embryonic development [29]. For instance, knockout of the *DUSP2* gene in mice led to the enhanced differentiation of TH17 cells and elevated secretion levels of pro-inflammatory cytokines, thereby exacerbating inflammatory symptoms in animal models of enteritis [30]. In yeasts, YVH1 serves as a conserved dual-specificity phosphatase involved in the growth, differentiation, and virulence of fungal pathogens affecting plants and animals [31,32]. Although the DSP domain contributes to human MAPK signaling regulation, for most cellular functions exerted by YVH1 in fungi, it primarily relies on the ZB domain [33]. Within the realm of flora, DUSPs are involved in various physiological processes, such as growth development and stress response, as well as pest and disease resistance [34]. Therefore, DUSP, being a crucial protease, exhibits diverse biological functions [35]. *AtDsPTP1* was the first bispecific protein phosphatase identified in higher plants, and experimental findings demonstrated its in vitro ability to effectively inhibit MAPK [36,37]. In plants, the role of *AtDsPTP1* extends beyond controlling the accumulation of abscisic acid (ABA) to include its involvement as a suppressor in signaling pathways related to osmotic stress. This discovery unveils the regulatory role of *AtDsPTP1* on pivotal signal transduction pathways in plants, particularly ABA signaling and osmotic stress response [38]. These observations suggest that *AtDsPTP1* plays a crucial role in the physiological processes of plants by influencing MAPK signaling pathways and participating in stress response mechanisms to adapt to environmental changes [39]. In cotton, *GhDsPTP1* serves as a negative regulator as a bispecific protein phosphatase within the salt stress response pathway. Through yeast two-hybrid screening, proteins interacting with *GhDsPTP1* were successfully identified, including the Ca^2+^ channel-attached protein *GhANN8*, which responds to stress signals [40]. The findings suggest that NaCl induces the expression of both *GhDsPTP1* and *GhANN8*, highlighting the significant regulatory role of *GhANN8* within the salt-tolerance pathway of cotton [41,42]. These discoveries offer significant perspectives for further understanding the molecular response mechanism of cotton towards stress [43].

DUSPs are heteroprotein phosphatases that possess dual functionality as tyrosine and serine/threonine phosphatases, facilitating the dephosphorylation of protein residues, thus allowing them to fulfill essential regulatory functions in diverse biological processes [44,45]. Despite the acknowledged significance of DUSP in biology, the origin and evolutionary history of the DUSP gene family remain unclear, with uncertainty surrounding its phylogenetic classification [46]. The primary objective of this study is to comprehensively identify the DUSP gene family in upland cotton, as no relevant information has been identified in upland cotton to date. This will facilitate an understanding of the diversity within this gene family and the determination of evolutionary relationships among its members. Additionally, the comprehensive identification of gene family members will contribute to the discovery of new potential functions or the revelation of known functional diversity. Subsequently, through functional identification, we ascertain the function of these genes and reveal their impact on phenotype, thus gaining a deeper understanding of their role and regulatory mechanisms in biological processes [47,48,49]. These efforts are intended to deepen our comprehension of this gene family and provide crucial information and a foundation for studying related biological processes.

## 2. Results

### 2.1. Identification and Basic Information of Cotton DUSP Phosphatase Family Members

Analyzing the whole-genome protein sequence information of cotton, 120 DUSP genes were detected within *G. arboreum, G. barbadense, G. hirsutum,* and *G. raimondii*. Among them, 32 GaDUSP genes, 42 GbDUSP genes, 29 GhDUSP genes, and 17 GrDUSP genes were identified (Supplementary Table S1).

The physicochemical analysis of proteins encoded by members of the DUSP family of *G. arboreum* showed that the amino acid count of the family proteins varied between 201 and 1255, and their relative molecular mass ranged from 22.81 to 141.35 kDa. Their theoretical isoelectric point ranged from 5.52 to 9.46, their instability coefficient from 37.65 to 60.67, their fat coefficient from 61.60 to 98.13, and their average isoelectric point values from −0.55 to 0.02. The average hydrophilicity (GRAVY) was −0.33. The majority of the genes were predicted to be situated within the cytoplasm and nucleus based on subcellular localization analysis (Supplementary Table S1).

A thorough examination of the physical and chemical characteristics exhibited by the proteins derived from individuals within this group of the DUSP family of *G. barbadense* was performed, and the proteins ranged in length from 138 to 960 amino acids and in relative molecular mass from 16.5 to 107.76 kDa. The theoretical isoelectric point range was 4.59 to 9.78, the instability coefficient was 37.05 to 63.79, the fat coefficient was 67.32 to 103.77, and the average isoelectric point value was −0.62 to 0.28. In addition, the average hydrophilicity (GRAVY) stood at −0.31. Subcellular localization predicted that most of the genes in this family were located in chloroplasts and nuclei. These results are detailed in Supplementary Table S1.

The physical and chemical analysis of the proteins encoded by members of the DUSP family in *G. hirsutum* revealed that the family proteins ranged in length from 219 to 935 amino acids and in relative molecular mass from 24.94 to 104.14 kDa. The theoretical isoelectric point range was 5.52 to 9.39, the instability coefficient was 37.72 to 62.36, the fat coefficient was 67.32 to 98.13, and the average isoelectric point value was −0.62 to −0.07. In addition, the average hydrophilicity (GRAVY) was −0.32. Based on the prediction of subcellular localization, most genes in this family were located in the cytoplasm and nucleus (Supplementary Table S1).

The characteristics and attributes of the proteins, in terms of their physical and chemical properties, encoded by members of the DUSP family of *G. raimondii* were analyzed; the length of the proteins of this family was observed to range from 264 to 876 amino acids, and the molecular weight ranged from 29.35 to 97.60 kDa. The potential isoelectric point range fell within 5.44 and 9.41, according to theoretical analysis; the instability coefficient was 40.77 to 63.96; the fat coefficient was 67.70 to 96.77; and the average isoelectric point value was −0.59 to 0.10. In addition, the average hydrophilicity (GRAVY) stood at −0.27. According to the subcellular localization, most of the genes in this family were located in the cytoplasm and nucleus. This set of analytical findings indicated that the members belonging to the DUSP family of cotton were all low hydrophilic proteins (Supplementary Table S1).

### 2.2. Distribution of DUSP Family Gene Chromosomes in Cotton

**Figure 1:**
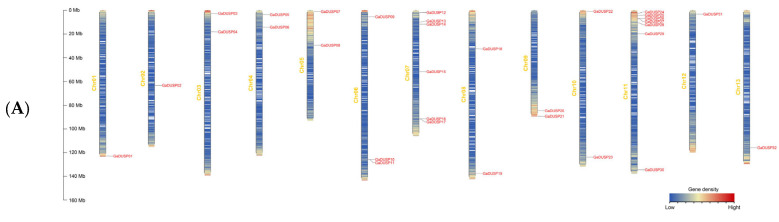

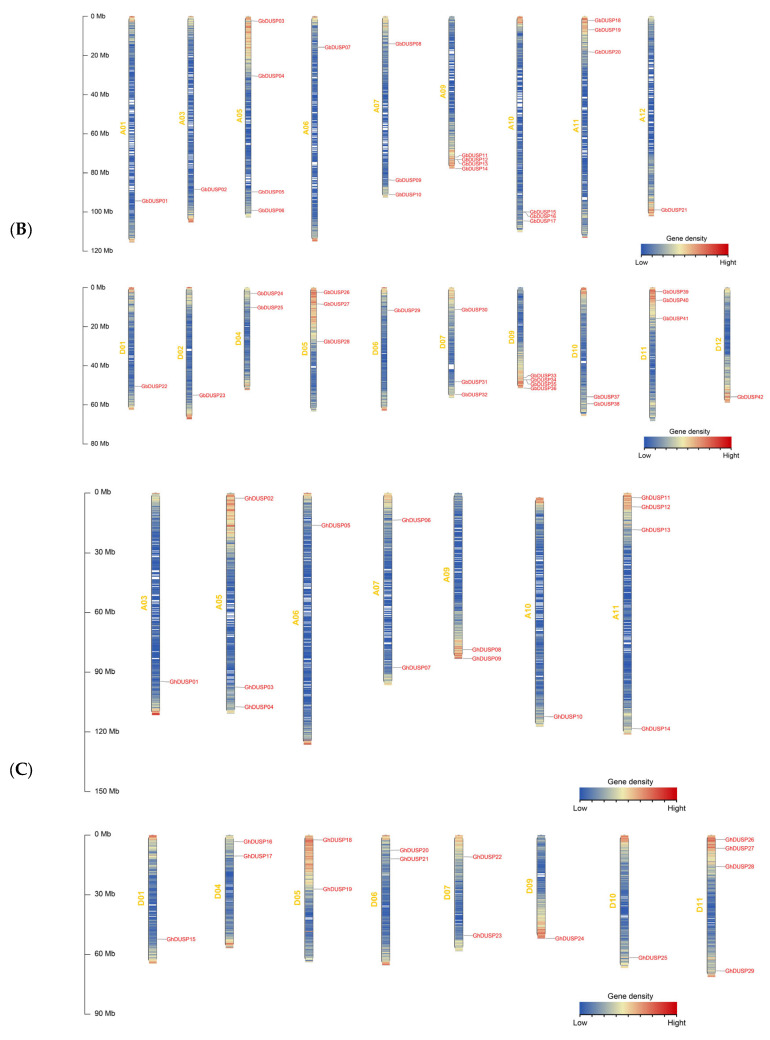

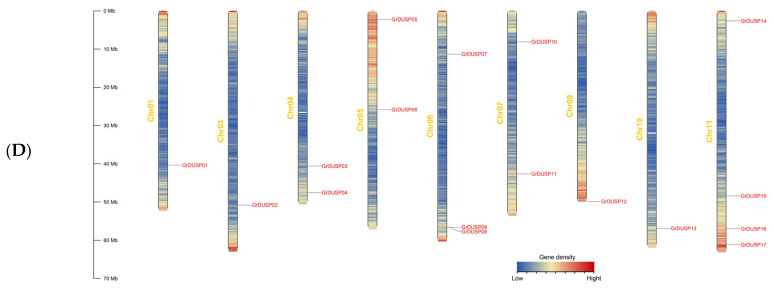
Chromosomal localization of DUSP family genes in *G. arboreum, G. barbadense*, *G. hirsutum,* and *G. raimondii.* (**A**) Location of *G. arboreum* gene on chromosome. (**B**) Location of *G. barbadense* gene on chromosome. (**C**) Location of *G. hirsutum* gene on chromosome. (**D**) Location of *G. raimondii* gene on chromosome.

We performed a precise mapping analysis to ascertain the specific positions of DUSPs on the corresponding chromosomes. Utilizing the upland cotton TM-1 protein gff3 file and Gene ID information, we employed the TBtools software (v1.098769) to map the chromosomal distribution of DUSPs. The analysis revealed a random distribution tendency of 120 DUSP genes across the chromosomes of *G. arboreum*, *G. raimondii, G. barbadense,* and *G. hirsutum*. This chromosomal map provides valuable insights into the spatial distribution of DUSP genes across various cotton species, facilitating further investigations into their potential impact on evolution and functional development (Figure 1).

In *G. arboreum*, we observed 32 genes randomly distributed across 13 chromosomes, and the quantity of DUSP genes varied between one and seven per chromosome. However, the lowest gene count was found in Chr01, Chr02, Chr12, and Chr13, while Chr11 had the highest gene count, resulting in a combined count of seven genetic elements.

In *G. barbadense*, the distribution of 42 DUSP genes was observed across 19 chromosomes, with each chromosome containing between one and four genes. Notably, chromosomes A05, A09, and D09 contained the most genes, each with four. Subgroups A and D had an equal number of genes, each with 21. However, no distribution of DUSP genes was observed on chromosomes A02, A04, A08, A13, D02, D08, and D13.

In *G. hirsutum*, 29 DUSP genes were randomly distributed across 15 chromosomes, and the range of the gene count per chromosome varied between one and four. A11 and D11 contained the most genes, with four. Subgroup A had 14 chromosomes, and subgroup D had 15 chromosomes, with one extra gene in subgroup D. However, no distribution of DUSP genes was observed on chromosomes A01, A02, A04, A08, D02, D03, and D08.

In *G. raimondii*, 17 DUSP genes were randomly distributed across nine chromosomes, and the range of the gene count per chromosome varied between one and four. The Chr11 chromosome contained the most genes, with four. However, no distribution of DUSP genes was observed on chromosomes A01, A02, A04, A08, D02, D03, and D08.

The results of this study indicated that the DUSP gene was relatively unevenly distributed in the chromosomes of the four species of cotton, with high gene density in some chromosomes and chromosome regions. This uneven distribution may reflect genetic variation in the course of evolution, resulting in the loss or duplication of genes on certain chromosomes.

### 2.3. Phylogenetic Assessment of DUSP Family Genes in Cotton

**Figure 2:**
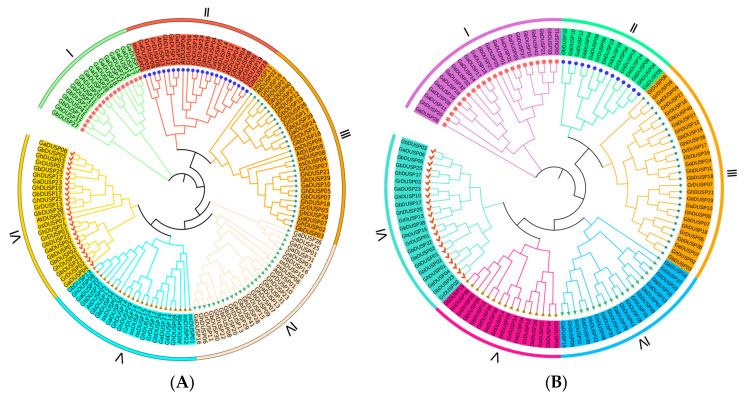
Phylogenetic relationships of DUSP family genes in plants. (**A**) Phylogenetic relationships of DUSPs in Arabidopsis, rice, *G. arboreum, G. raimondii, G. barbadense,* and *G. hirsutum*. (**B**) Phylogenetic relationships of DUSPs in four major cotton varieties. (**A**) The outer circles are marked with green, red, brown-yellow, light pink, blue, and yellow, representing groups I, II, III, IV, V, and VI, respectively. (**B**) The outer circles are marked with purple, green, brown-yellow, blue, light purple, and light blue, representing groups I, II, III, IV, V, and VI, respectively.

To explore the evolutionary aspect of connections between plant members of the DUSPs, a comparison was conducted on the amino acid sequences of cotton DUSP with those found in Arabidopsis and rice, constructing a phylogenetic tree containing 144 protein sequences using the Maximum Likelihood method (ML) (Figure 2). These sequences included 120 DUSP proteins in cotton, 9 in Arabidopsis, and 15 in rice. The phylogenetic tree showed that the DUSPs can be separated into six branches with randomly distributed members, with the fewest genes in evolutionary branch I (about 17) and the most genes in evolutionary branch VI (about 28).

In each evolutionary branch, the DUSP proteins have corresponding homologues, suggesting a close relationship among the DUSP proteins within these plant species. The number of DUSP genes per subgroup of the allotetraploid cotton species is essentially double that of each subgroup of the diploid cotton species. The DUSPs of *G. arboreum, G. raimondii*, *G. barbadense*, and *G. hirsutum* have corresponding homologues in almost every clade, indicating a close genetic relationship between them. The number of protein phosphatase genes with bispecificity in each subpopulation of *G. hirsutum* and *G. barbadense* is approximately twice as high as that in each subpopulation of *G. arboreum* and *G. raimondii*, aligning with the findings from previous analyses and corresponding to the evolutionary relationship observed among cotton species. It is possible that the conservation of bispecific protein phosphatase family genes during the evolution of cotton could be attributed to evolutionary selection following the hybridization of diploid cotton to form allotetraploid cotton. While the number of individuals in the initial subgroup is limited, they have been preserved throughout the evolutionary process of cotton, indicating their potential significance in various biological mechanisms.

We noticed that, in the phylogenetic trees, GhDUSP and GbDUSP gene pairs were consistently grouped together, indicating a possible occurrence of gene duplication. The number of bispecific protein phosphatase genes in tetraploid cotton (*G. hirsutum* and *G. barbadense*) and diploid cotton (*G. arboreum* and *G. raimondii*) further confirmed that GhDUSP and GbDUSP (*G. hirsutum* and *G. barbadense*) are the main genes of *G. arboreum* and *G. raimondii* as a result of hybridization.

### 2.4. Motifs and Gene Structure of DUSP Family Genes in Cotton

**Figure 3:**
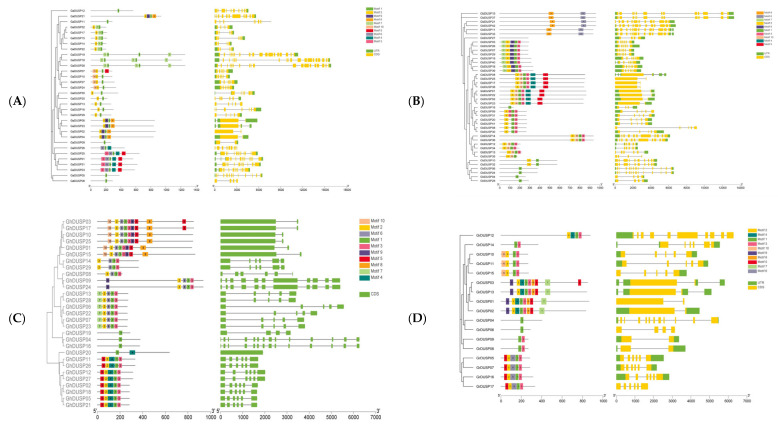
Phylogenetic analysis of DUSP motifs and gene structure in cotton. (**A**) Phylogenetic tree of *G. arboreum*. (**B**) Phylogenetic tree of DUSPs of *G. barbadense*. (**C**) Phylogenetic tree of *G. hirsutum*. (**D**) Phylogenetic tree of *G. raimondii*.

To further understand the structural evolution of DUSP family genes in four major cotton varieties, we conducted a systematic analysis of the gene structure and conserved motifs (Figure 3). In *G. arboreum*, similar genes clustered in the same group of developmental trees, and the count of exons per gene ranged from one (*GaDUSP12*, *GaDUSP11*, *GaDUSP09*, *GaDUSP05*, and *GaDUSP08)* to five (*GaDUSP18*, *GaDUSP28*, *GaDUSP25*, *GaDUSP31*, and *GaDUSP03*). In the majority of instances, two genes in a gene pair had similar exon–intron structures, such as *GaDUSP10/27*, *GaDUSP10/27*, *GaDUSP23/06,* etc. In *G. barbadense*, similar genes were clustered in a set of data that formed identical developmental trees, and the number of exons per gene ranged from one (*GbDUSP16*, *GbDUSP06*, *GbDUSP24*, etc.) to six (*GbDUSP05*, *GbDUSP25*, and *GbDUSP01*). Typically, in the majority of instances, two genes in the gene pair had a similar exon–intron structure, such as *GbDUSP10/32*, *GaDUSP11/33*, *GaDUSP14/36*, etc. In *G. hirsutum*, similar genes were clustered in the same set of developmental trees, and the number of exons per gene ranged from one (*GhDUSP19*, *GhDUSP04*, *GhDUSP16,* etc.) to nine (*GhDUSP03* and *GhDUSP17*). In most cases, two genes in the gene pair had similar exon–intron structures, such as *GhDUSP09/24*, *GhDUSP14/29*, etc. In *G. raimondii*, similar genes were clustered in the same set of developmental trees, and the number of exons per gene varied from one (*GrDUSP04* and *GhDUSP06*) to nine (*GrDUSP03*), and two genes in the gene pair had similar exon–intron structures, such as *GhDUSP09/08*, *GhDUSP05/07*, etc. There were also some genes that constituted gene pairs; however, their gene structure and gene length were slightly different. There were variations in the exon count across different clades; however, it was observed that most GhDUSP members within the same clade shared a consistent exon–intron structure. This suggests a strong correlation between the genetic makeup of GhDUSP genes and their evolutionary phylogeny, highlighting the intricate nature of gene architecture.

Analyzing the sequences of the proteins of DUSP family members in four cotton varieties using the online tool MEME, we obtained 10 motifs, and the proteins with the same motif composition showed a tendency toward preferential aggregates. As shown in Figure 3, most DUSP members that were clustered on the same clade, especially closely related members, had the same motifs. There were clear differences between the genes, as well as significant differences in the structure of the two genes, suggesting that they may have lost some function during evolution.

### 2.5. Exploring the Collinearity among Members of the DUSP Family in Cotton

**Figure 4:**
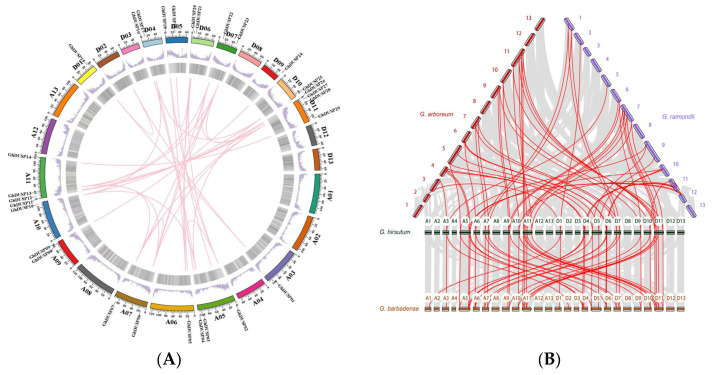
Duplicated DUSP gene pairs identified in cotton. (**A**) Collinearity analysis of DUSPs in *G. hirsutum* to study its structural conservation and potential evolutionary relationship. (**B**) Multiple homolinear analyses were performed to elucidate the lineal homology relationships between DUSP genes in cotton. The analysis used chromosomal visualizations of different cotton varieties with different colors.

To investigate the evolutionary relationship of DUSPs in *G. arboreum*, *G. raimondii*, *G. barbadense*, and *G. hirsutum*, we performed a collinearity analysis, with upland cotton selected as the core species, and we constructed an intraspecies collinearity diagram (Figure 4). In the analysis of collinearity within the same species, *G. hirsutum* was examined, and we observed gene distribution on 15 chromosomes with 37 tandem repeats and significant collinearity between subgroup A and subgroup D. These findings suggest that there were chromosome duplication and tandem duplication events in upland cotton, while no gene deletions were detected. The presence of collinearity indicates potential structural changes at the chromosomal level during gene evolution, providing valuable insights for further exploration into DUSP gene evolution.

### 2.6. Examination of Regulatory Elements in the GhDUSPs

**Figure 5:**
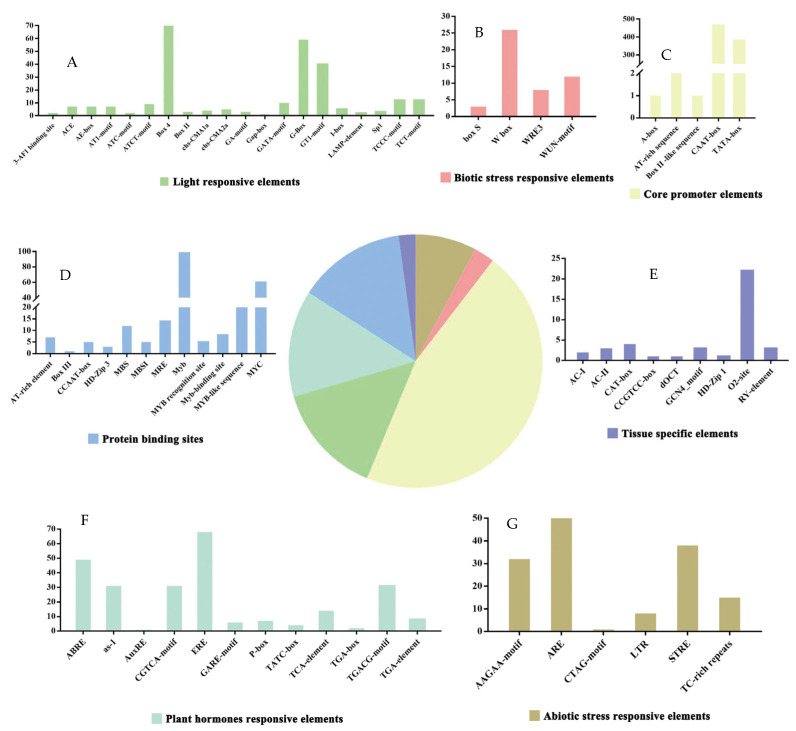
Cis-acting element analysis of GhDUSP family genes. In the sequential action element analysis, light-responsive elements (**A**) refer to gene-regulatory elements that respond to light changes. These elements exist in the plant genome and can respond to changes in light and regulate the expression of related genes. Biotic stress-responsive elements (**B**) refer to gene-regulatory elements that respond to biological stress (such as pathogens, pests, etc.). Core promoter elements (**C**) refer to DNA-sequence elements located near the transcription start point that play an important role in gene transcription initiation and regulation. Protein-binding sites (**D**) refer to specific regions where proteins bind to other molecules (usually another protein, DNA, RNA, etc.). The specificity and affinity of protein-binding sites are crucial for regulating intracellular signal transduction, gene expression, metabolic pathways, and other biological processes. Tissue-specific elements (**E**) refer to gene-regulatory elements that function in specific tissues or cell types. Plant hormone-responsive elements (**F**) refer to gene-regulatory elements that respond to hormone signals in plants. Abiotic stress-responsive elements (**G**) refer to gene-regulatory elements that respond to non-biological stress (such as drought, high temperature, salt stress, etc.).

The potential roles of proteins encoded by downstream genes can be revealed through the prediction and analysis of cis-acting elements present in promoter regions. Our prediction and analysis of cis-acting elements in 29 GhDUSP gene-promoter regions revealed a large number of core elements, including TATA-box and CAAT-box, as well as multiple photoresponsive elements (e.g., G-box, AE-box, MRE, Sp1, etc.). Hormone-related cis-acting elements were abundant and diverse, including gibberellin-related elements (e.g., TATC-box, GARE-motif, and I-box), ABREE, auxin (TGA-element), salicylic acid (TCA-element), and methyl jasmonate-related elements (e.g., CGT, Ca-motifs, Ga-motifs, and Gare-motifs). The cis-acting elements related to stress mainly included drought-related elements (MBS and MYB), low-temperature-related elements (LTR), anaerobic inducible-related elements (ARE), hypoxic inducible-related elements (GC-motif) and defense and stress-response elements (TC-rich repeats). In addition, cis-acting elements associated with the meristem (such as CAT-box and CCGTCC-box) were also found, and some genes contained cis-acting elements related to the regulation of zein metabolism (O2-site). These findings imply that the GhDUSP genes might contribute to the growth and development of plants, as well as to their ability to adapt to environmental challenges (Figure 5).

### 2.7. Tissue-Specific Examination of Gene Expression within the GhDUSPs

**Figure 6:**
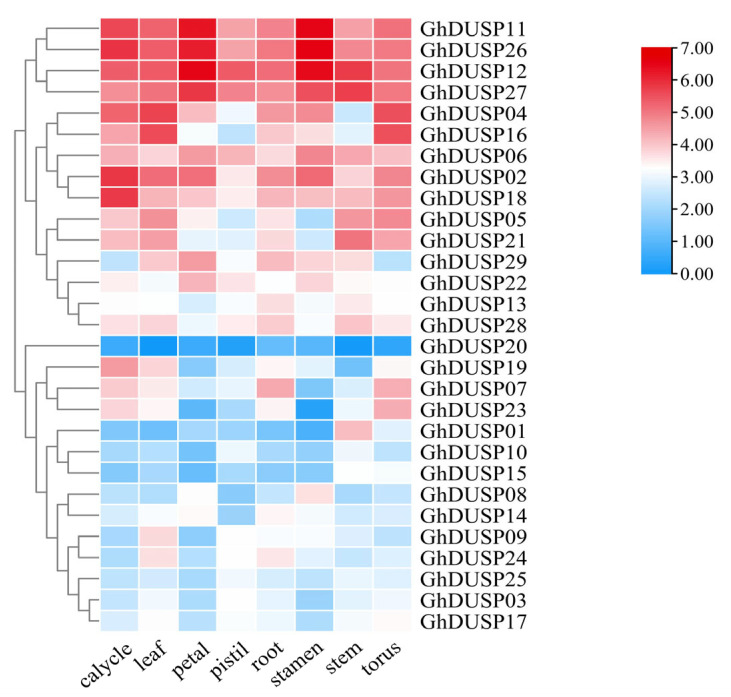
Tissue-specific expression investigation of GhDUSP genes utilizing transcriptome data.

When analyzing the RNA-seq data of DUSP genes in upland cotton downloaded from the CottonFGD (https://cottonfgd.org/ accessed on 20 August 2023) website (PRJNA248163), we found that 29 GhDUSP family genes exhibited two distinct expression patterns and showed different degrees of expression differences in different tissues (Figure 6). Among them, 15 genes belonged to the first expression mode, which was mainly highly expressed in petals, leaves, stems, stamens, and receptacles, while the expression level was lowest in the pistil. In addition, 14 genes belonged to the second expression mode, and their expression levels varied greatly among different tissues, being mainly present in the calyx and stamen. The majority of the genetic material had the highest expression level in the stamen. This result suggests that GhDUSPs have specific gene expression patterns and may be involved in more complex biological functions.

### 2.8. Exploring the Impact of Various Stressors on the Expression Patterns of GhDUSPs

**Figure 7:**
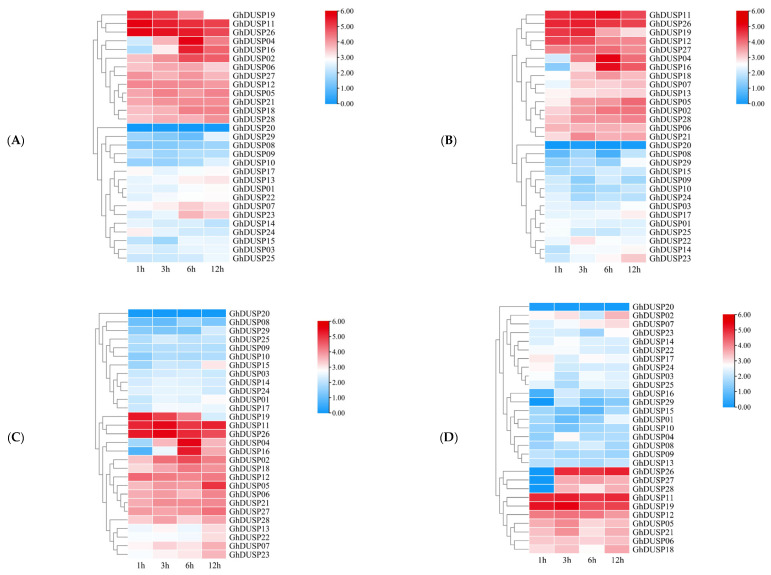
Expression analysis of GhDUSPs under stress treatment. ((**A**) Cold, (**B**) Drought, (**C**) Heat, and (**D**) Salt.

To gain a deeper insight into how GhDUSPs respond to abiotic stress, we performed a study analyzing the gene expression profiles of these genes in response to different stressors, including low temperature, high temperature, salt exposure, and PEG (Polyethylene Glycol) treatment. Additionally, we visually represented the expression levels using heat maps (Figure 7). Some GhDUSPs showed similar expression levels under multiple abiotic stresses, while others showed strong induction and were significantly differentially expressed under multiple stresses. Specifically, cold treatment resulted in significant changes in the expression of 13 genes of the first class (Figure 7A), suggesting that the upregulation of these genes was triggered through exposure to low temperatures and could potentially contribute significantly to the adaptation of upland cotton in cold environments. In PEG stress, 15 genes were upregulated, and they reached a peak value at 12 h, suggesting a potential involvement of these genes in enhancing cotton’s ability to withstand drought conditions (Figure 7B). Under heat stress, 17 genes were significantly upregulated, peaking at 6 h and continuing to increase at 12 h, suggesting their potential involvement in conferring heat resistance in upland cotton (Figure 7C). Following salt stress, the expression of 10 genes in the third branch was upregulated, with two genes exhibiting high expression at 1 h and a continuous increase until reaching maximum levels at 12 h. This indicates that these identified genes may contribute to salt tolerance in cotton (Figure 7D). In general, multiple genes showed high expression under different abiotic stresses, indicating that they may have synergistic regulatory effects under drought, salt, heat, and cold abiotic stresses. Some genes showed different dominant expressions under different stress conditions, indicating that the responses of GhDUSP members to specific stress types were different. Overall, the expression levels of about half of the GhDUSP genes changed significantly under different stress conditions, emphasizing their important role in the drought, salt, heat, and cold abiotic stress responses of upland cotton.

### 2.9. Expression Analysis of GhDUSPs in Verticillium Dahliae

**Figure 8:**
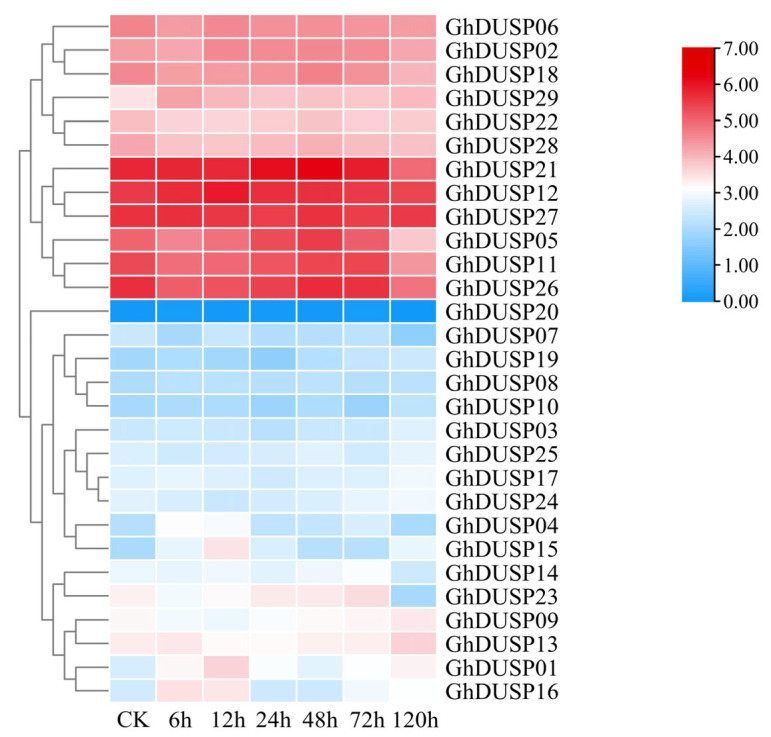
Investigation of the expression patterns of GhDUSPs under the influence of *Verticillium wilt* stress.

To explore the function of GhDUSPs in the participation of *Verticillium wilt* in abiotic stress, the RNA-seq data of inoculated *Verticillium wilt* (PRJNA248163) were used for analysis, and these *GhDUSP* genes aggregated into three expression patterns, among which, six genes in the second branch showed a high expression trend *(GhDUSP21, GhDUSP12, GhDUSP27*, *GhDUSP05*, *GhDUSP11*, and *GhDUSP26)*. Within this group, the levels of expression for three specific genes were examined (*GhDUSP05*, *GhDUSP11*, *GhDUSP14*, *GhDUSP26*), which fluctuated highly between 0 and 72 h and began to decrease at 120 h; these genes may be involved in the stress response of *Verticillium wilt* in cotton. The expression level of *GhDUSP14* increased after being induced by pathogens; the peak was observed at 72 h, with a decrease starting from 120 h, suggesting that this particular gene could potentially contribute to the later phase of infection resistance against *Verticillium wilt* in cotton (Figure 8).

### 2.10. Detection of Verticillium wilt GhDUSPs Using qRT-PCR

**Figure 9:**
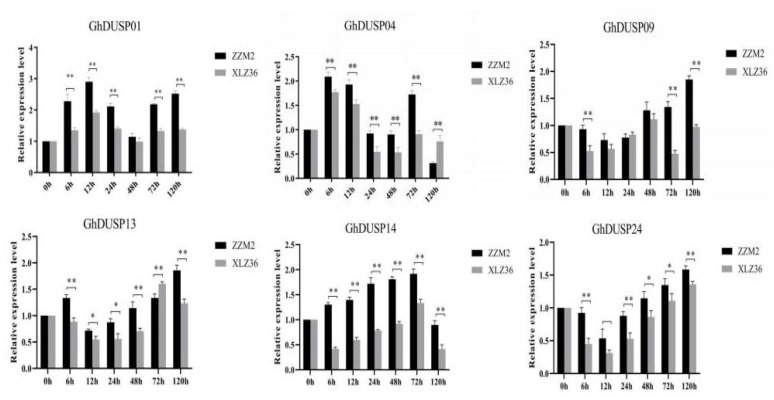
Gene expression pattern of GhDUSPs in upland cotton triggered by *Verticillium wilt*. Each experiment was conducted with three technical replicates and three biological replicates. The error bars represent the mean values from three technical replicates ± standard errors. Statistically significant differences compared to the control group are denoted by * *p* < 0.05, ** *p* < 0.01.

The patterns of expression of GhDUSP genes were compared between Zhongzhimian 2 (resistant variety) and Xinluzao 36 (susceptible variety), based on the transcriptome database of upland cotton. The findings indicated that the levels of gene expression of these GhDUSP genes changed when induced by *Verticillium wilt*, suggesting that they play a role in the host defense against *Verticillium wilt*. Notably, the qRT-PCR experimental findings aligned with the GhDUSPs observed in the transcriptome data. Therefore, we hypothesized that six of these genes (*GhDUSP01*, *GhDUSP04*, *GhDUSP09*, *GhDUSP13*, *GhDUSP14*, and *GhDUSP24*) were associated with *Verticillium wilt* resistance. We used the qRT-PCR real-time fluorescence quantitative technique to analyze the differential expression of these six GhDUSPs in Zhongzhimian 2 and Xinluzao 36 under the pathogenic stress of *Verticillium wilt*. All of these genes changed significantly at different time points after stress response (Figure 9), which further indicated that these GhDUSP genes induced by *Verticillium wilt* were involved in the parasite process of *Verticillium wilt* in upland cotton. Specifically, we observed that, in these six candidate genes, the expression level of *GhDUSP14* was slightly higher, and the expression level continued to increase after the pathogen stress but began to decline at 120 h. This expression pattern is consistent with the typical characteristics of cotton induced by abiotic stress. Second, based on the results of the previous transcriptome sequencing RNA-seq and genome-wide association analysis of GWAS, we identified *GH_A11G3500* (*GhDUSP14*), a gene located on chromosome A11 and detected in various instances of associated environmental data. Taking this information into account, we tentatively speculated that *GH_A11G3500* (*GhDUSP14*) may participate in the defense against *Verticillium wilt*. Therefore, we chose this gene as the focus of our subsequent studies (Figure 9).

### 2.11. Functional Verification of GH_A11G3500

**Figure 10:**
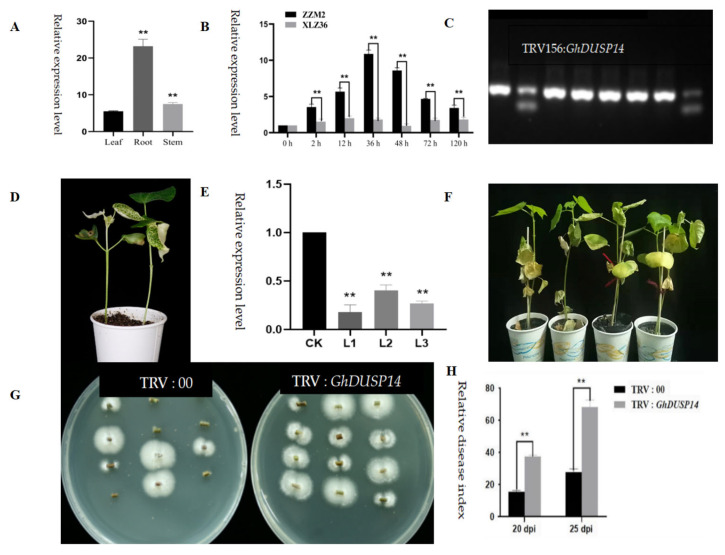
Silencing GH_A11G3500(*GhDUSP14*) impaired the resistance of cotton to *V. dahliae*. (**A**) To compare the expression levels of target genes in root, stem and leaf; (**B**) The expression levels of target genes in the root tissues of Zhongzhimian 2 and Xinluzao 36 were analyzed; (**C**) Construction of target gene silencing vector; (**D**) Silent plants showed albino phenotype; (**E**) Differential expression of target genes in silenced and control plants. CK was the control; L1, L2, and L3 represent three biological replicates, each containing 30 cotton plants; (**F**) The phenotype of the target gene silenced plants at 15 d and 25 d (the two pots on the left represent 15 d and the two pots on the right represent 25 d); (**G**) Fungal recovery experiments. Stem segments of TRV:00 and TRV:*GhDUSP14* plants were incubated on PDA medium at 25 °C. The samples were photographed 3 d later. Note: TRV:00 indicates an empty vector; TRV:GhDUSP14 indicates the silenced *GhDUSP14*; (**H**) Relative disease index of TRV:*GhDUSP14* at 15 d and 25 d. The mean ± standard error of the three technique replicates is depicted. The error bars represent the mean values from three technical replicates ± standard errors. Statistically significant differences compared to the control group are denoted by ** *p* < 0.01.

The choice of *GH_A11G3500* (*GhDUSP14*) as the focal gene was made after conducting a thorough examination of the RNA-seq data and GWAS, which led to the identification of a potential candidate gene. First, we conducted initial fluorescence quantitative analysis of the target gene in root, stem, and leaf. Upon analysis, we found that the gene was expressed at relatively high levels in the root tissues (Figure 10A), so we chose root tissues as the sample for the subsequent research.

Fluorescence quantitative differential expression of this gene was detected in the root, stem, and leaf tissues of Zhongzhimian 2 and Xinluzao 36 at 0 h, 2 h, 12 h, 36 h, 48 h, 72 h, and 120 h after infection with *Verticillium wilt.* It was expressed as follows: *GhDUSP14* was higher in the root tissues of the plant, 4.2 times that of the leaves and 2.5 times that of the stems of the same segments; so, the root tissues were selected for a further detailed analysis (Figure 10B). The expression levels of *GH_A11G3500* at 0 h, 2 h, 12 h, 36 h, 48 h, 72 h, and 120 h in the root tissues of Zhongzhimian 2 were measured. The findings indicated that *GH_A11G3500* expression reached its highest level at 36 h in the root tissues of Zhongzhimian 2, which was nearly 11 times that at 0 h. The expression of *GH_A11G3500* began to decrease at 48 h (Figure 10B). However, in susceptible varieties, the expression of this gene was significantly different from that in resistant varieties; in susceptible varieties, the expression of this gene tended to be consistent in each time period, and the expression level was low. The gene was highly expressed in the root 36 h after infection, which is consistent with the transmission path of *Verticillium wilt* into cotton plants; that is, it first enters through the root tissue; enters the vascular bundle of the stem, causing the vascular bundle to fail to supply nutrients to the plant; and then enters the leaf tissue of the plant, causing the leaves to yellow and wilt, affecting the growth of the plant. This strongly suggests that the involvement of this gene in regulating cotton’s resistance to *Verticillium wilt* is highly probable.

The root of Zhongzhimian 2 inoculated with *Verticillium wilt* for 48 h were used as the material for cloning *GH_A11G3500* (*GhDUSP14*). The size of the cloned vector band matched the size of the target gene fragment, and the sequencing results comparison indicated successful cloning (Figure 10C). VIGS experiments were performed, and the chlorophyll in the new leaves of the PDS control plants gradually decreased 10 days after injection, indicating that VIGS injection was successful (Figure 10D). Through the identification of the target gene’s expression level, it was found that the expression of *GH_A11G3500* was significantly decreased in the silenced plants (Figure 10E), further confirming the successful interference with the target gene.

The unloaded control plants also showed symptoms of disease after 25 days of inoculation with *Verticillium wilt*, but their growth was relatively normal. In contrast, plants silenced with the target gene showed wilting leaves, a watermelon-rind shape, and, eventually, yellowing and shedding. According to the disease statistics, the relative disease index of the control plants was 38.94, while that of the plants silenced with *GH_A11G3500* was 76.82, indicating that the cotton plants silenced with *GH_A11G3500* were more sensitive to *Verticillium wilt* (Figure 10F). This strongly indicates that *GH_A11G3500* has a significant impact on the defense mechanism of upland cotton against pathogen invasion.

In order to evaluate the content of pathogens in the control plants and silenced plants, we performed a profile treatment of cotton stems and carried out the isolation and recovery test of pathogens (Figure 10G). The results showed that, in the stem segment of the control plants with TRV:00, the number of single colonies of *Verticillium wilt* was small, while in the silenced plants with TRV: *GH_A11G3500*, all colonies of *Verticillium wilt* were isolated. This indicated that, in the silenced plants, the degree of infestation was more profound, while in the control plants, the degree of infestation was relatively low. Therefore, the recovery test of *Verticillium wilt* in the stem segment intuitively demonstrated the degree of pathogen infection in the two treated plants. The disease conditions of the TRV: *GH_A11G3500*-silenced plants and TRV:00 control plants were statistically analyzed. The results showed that, at 20 d and 25 d after inoculation, the disease index of the cotton plants subjected to *GH_A11G3500* gene silencing exhibited a notably elevated level compared to the control group without any genetic modification, suggesting an increased susceptibility of the cotton plants towards *Verticillium wilt* stress following the silencing process (Figure 10H). This further proved that *GH_A11G3500* plays an important role in the resistance of plants to the infection of *Verticillium wilt*.

## 3. Discussion

### 3.1. Phosphorylation of DUSP Family Genes

Protein phosphatases are widely involved in the regulation of eukaryotic cells. Plant protein phosphatases have been shown to be ubiquitous in cell signal transduction [50], and systematic studies have been conducted in multiple model organisms—including yeast, Arabidopsis, rice, cotton, and humans—revealing the important roles of many protein kinases in plant defense, metabolism, and signal transduction [51]. In recent years, plant protein phosphatase studies have also identified protein phosphorylation roles in multiple cell signal transduction pathways, providing insights into plant cell regulation [52]. There are more than 1000 protein kinase coding sites in the Arabidopsis genome, compared to about 300 for protein phosphatases [53]. These protein phosphatases include Ser/Thr phosphatases, Tyr phosphatases, and bispecific phosphatases, where DsPTPs regulate plant growth and development under stress conditions [54]. For example, mutations in the Mkp1 gene cause plants to respond differently to UV and salt stress [55]. Future studies need to further explore the effects of DSP on other signaling pathways, especially through indirect effects on changes in MAPK activity or direct effects on other mediators [56]. There is no evidence for the direct binding and dephosphorylation of non-MAPK targets via conventional DSP-MKP modules, suggesting that DSP may influence multiple signaling pathways in unusual ways, extending its diphosphatase activity to a wide range of target proteins and even RNA [57]. For pathways known to have crosstalk, the DSP family may have indirect effects, and future studies need to focus on these complex interactions to better understand the mechanisms of DSP in cell signal regulation [58].

### 3.2. Basic Analysis of DUSP Families in Four Major Cotton Species

We identified the DUSPs gene family on a genome-wide scale in cotton, which is a first step towards understanding its role in disease-resistance mechanisms. Through bioinformatics methods, we systematically mined the sequence information of all DUSPs genes in the cotton genome, including the number, distribution, structural characteristics, evolutionary relationships, cis-acting elements, and their stress responses under biological and abiotic stresses. In this process, we found that, first, in the promoter analysis, the DUSPs gene can regulate key proteins in the signal transduction pathway through dephosphorylation, thereby affecting the efficiency and direction of signal transduction. This helps cotton to respond to pathogen infection in time and initiate corresponding resistance response. DUSPs genes can also influence the activity of transcription factors, thereby regulating the expression of disease resistance-related genes, and regulate the activity of disease course-related proteins through dephosphorylation. These proteins play a direct role in the disease resistance of cotton, such as antimicrobial peptides, cell wall-strengthening proteins, etc. [59,60]. By regulating the activity of these proteins, the DUSPs gene can enhance the resistance of cotton to pathogens. This not only contributes to a comprehensive understanding of the diversity of DUSPs gene families but also provides basic data for subsequent functional studies.

### 3.3. Potential Role of DUSPs Gene in Disease Resistance of Upland Cotton

Limited understanding and knowledge gaps exist regarding the dephosphorylation of bispecific protein phosphatases in plants. Recent studies have highlighted the crucial role of the phosphatase family in regulating plant response to abiotic stress, which has become a focal point of current research. Plants adapt to harsh environments by modulating the function of the phosphatase family in their response to abiotic stress [61]. Reversible phosphorylation equilibrium catalyzed by protein kinase and protein phosphatase is considered a mechanism through which plants sense abiotic stress, serving as a direct molecular “switch” that regulates downstream gene expression and cell metabolic activity related to stress [62]. Despite potential functional redundancy affecting the analysis of their physiological functions, DUSPs can still provide a robust research network if targeted for pathogen interactions in plant immune response assays [63].

In this study, we once again demonstrated that DUSP genes may influence resistance protein activity by regulating their phosphorylation state, thereby impacting cotton’s resistance to pathogens. By comparing GhDUSPs’ expression patterns and functionally identifying *GH_A11G3500* in resistant and susceptible varieties, we speculated on the relationship between GhDUSP genes and cotton resistance. The genome-wide identification of the GhDUSPs provides a new candidate gene resource for breeding disease-resistant cotton.

Through comprehensive study and utilization of these genes, new cotton varieties with superior disease resistance can be developed. For instance, precise editing using gene-editing technology can enhance disease resistance in GhDUSPs. Alternatively, optimizing cotton’s disease resistance response can be achieved by regulating GhDUSP gene expression patterns. Furthermore, GhDUSPs can serve as molecular markers for disease-resistant breeding programs and aid in selecting high-resistance cotton varieties.

## 4. Materials and Methods

### 4.1. Gene Members Belonging to DUSPs in Cotton were Identified

In this study, CottonGen was used to investigate *G. hirsutum* (CRI), *G. barbadense*, (NAU), *G. arboretum*, (CRI), and *G. raimondii* (JGI) (https://cottonfgd.org/ accessed on 20 August 2023). We downloaded the Arabidopsis AtDUSP protein sequence information from the Arabidopsis AIR database. We downloaded the seed file of the DUSP protein from the Pfam database and identified the amino acid sequence containing the DUSP conserved domain (E-value < 0.0001) using the hmmsearch tool in HMMER3.0 software. We removed the non-phosphorylated region of the gene, obtained the cotton DUSP amino acid sequence through the Pfam CD, and used the NCBI database for conservative domain confirmation; then, incomplete sequences of the conservative domain were removed to obtain DUSP family members. The ExPASy-ProtParam tool was utilized to analyze the physical characteristics of the proteins derived from members of the DUSP family.

### 4.2. Localization of DUSP Family Genes on Chromosomes

The genes of cotton were extracted from the gff annotation file of the genome. The chromosomal physical location information of the DUSPs was obtained, and the corresponding Gene ID was determined. This information, as well as information about the cotton genome chromosome length, was used in Mapchart 2.2. software and Adobe Illustrator CS6 software v2020 to map the chromosomal distribution of the genes.

### 4.3. Development and Evaluation of a Phylogenetic Tree for the DUSP Gene Family in Cotton

The Arabidopsis DUSP protein sequences were obtained from the TAIR website, and the genome annotation information was obtained for the DUSP rice protein sequence from (http://rice.plantbiology.msu.edu/ accessed on 20 August 2023). The Pfam website was used to confirm the Arabidopsis and rice DUSP protein domain structures. Then, MEGA7.0 software was used to perform phylogenetic analysis on the protein sequence of cotton DUSP, adopting the neighbor-joining method to select pairwise deletion and setting the bootstrap value to 1000. We completed the construction of the phylogenetic tree system. Finally, we used TBtools to visualize and present the phylogenetic tree.

### 4.4. Analysis of Conserved Patterns and Genetic Organization in the DUSPs of Cotton

The location information of DUSP gene introns and exons of cotton was extracted using cotton genome data. The protein sequences that were identified were subsequently utilized in the online tool MEME Suite 5.1.0 (https://meme-suite.org/ accessed on 20 August 2023) for conserved motif analysis. In this analysis, the motif number was set to 10, and other parameters, such as the length, were set to their default values. At the same time, the gene structure information of DUSP genes was acquired through downloading the genome gff3 annotation file from the cotton database and entering the annotation file into the TBtools software.

### 4.5. Exploring the Collinearity of DUSPs in Cotton

The duplication events of the DUSP gene family in land wool and cotton were examined using MCScanX (V1.1) software to effectively minimize redundancy. Next, the data were visualized using the Tbtools software.

### 4.6. Prediction of GhDUSPs’ Cis-Acting Components

The upstream 2000 bp sequence of the start codon of the DUSP gene in upland cotton was extracted from CottonFGD (https://cottonfgd.org/, accessed on 23 August 2023), using TBtools software, as the promoter region. Then, we used the Plant CARE database to determine the GhDUSP gene promoter region related to cis-element prediction.

### 4.7. Expression Analysis of GhDUSPs in Cotton under Different Tissues and Stresses

We acquired transcriptome sequencing data from the NCBI SRA database for eight different tissues—namely the root, stem, leaf, pistillate, stamen, calyx, petal, and receptacle (PRJNA248163)—as well as four types of stress (i.e., cold, heat, drought, and salt stress). The SolexQA platform was used for data quality control; TopHat2 software v2.1.0 was used for comparison; and Cufflink was used for transcript splicing, calculation of the FPKM value, calculation of the gene expression quantity, and the search for differentially expressed genes. Gene expression screening was conducted using FPKM > 1 as the criterion. The transcriptome data underwent log2(FPKM + 1) normalization. The TBtoolsv1.105 software was utilized to generate an expression heat map for members of the GhDUSP family.

### 4.8. Examination of the Disease Resistance Expression Patterns in GhDUSPs

Based on the RNA-seq data, an analysis was conducted on the genes that exhibited differential expression in upland cotton when subjected to *Verticillium wilt* stress. GhDUSP family members were selected, and the differentially expressed genes, responding to the stress of *Verticillium wilt*, were screened. Subsequently, after conducting hierarchical cluster analysis with the assistance of the TBtools software (V1.098769), heat maps were drawn of GhDUSP family members’ expression patterns at different time points. In addition, reconstructed phylogenetic trees were utilized to unveil the evolutionary connections among these genes.

### 4.9. RNA Extraction and qRT-PCR

In the experiment, Zhongzhimian 2 and Xinluzao 36 were cultivated in a cotton cultivation room using conventional cultivation methods and were supplied with water every two days. When two true leaves were grown, the plants were inoculated with *Verticillium wilt* (Vd592) using the root grafting method. Afterward, samples of tissue from the root, stem, and leaves were gathered at various intervals (0 h, 3 h, 6 h, 12 h, 24 h, and 36 h) and preserved using liquid nitrogen. RNA extraction was performed using an RNA extraction kit, and the obtained RNA was reverse transcribed for cDNA backup. Primer 5.0 software was utilized to create primers targeting specific regions of the DUSP gene sequence, using the cDNA data for the GhDUSP gene as a reference (Supplementary Table S3). The double-chain chimeric fluorescent dye method (SYBR Green I) was used to perform qPCR tests, with *GhUBQ7* as the internal reference gene. Two methods were used, including 2^−ΔΔCt^, to calculate the gene expression, and the treatment group/control group was used as the relative expression amount of each gene. There were three technical replicates and three biological replicates for each experiment.

### 4.10. Silencing the Target Gene GhDUSP14

We utilized the Virus-Induced Gene Silencing (VIGS) technique to investigate the potential role of *GhDUSP14* in providing resistance against *Verticillium wilt* in upland cotton. The genetic sequence of *GhDUSP14* CDS was incorporated into the TRV:*GhDUSP14* vector, and the silencing-vector TRV:*GhDUSP14* was constructed by inserting the specific *GhDUSP14* gene fragment into the restriction site of the TRV:*GhDUSP14* vector. Then, we transmuted TRV:*GhDUSP14* into GV3101. In the experiment, we initially selected the resistant variety Zhongzhimian 2 for cultivation. Once the seedling’s two cotyledons were fully unfolded, we used a 1 mL syringe needle to create two small punctures on the lower epidermis of the cotton cotyledon. Subsequently, we injected the TRV:00 and TRV:*GhDUSP14* solution into the plant (Figure 10). The bacterial solution completely filled the leaf and was cultured under alternating conditions of light for 8 h and darkness for 16 h. Upon observing an albino phenotype in the positive control plants, we randomly chose true leaves from the empty carrier cotton, as well as the plants with the silenced target gene. To assess the target gene silencing efficiency, cDNA obtained from the root tissue served as a template, with *GhUBQ7* acting as the internal reference gene. We utilized qRT-PCR to measure the TRV:*GhDUSP14* expression levels relative to *GhUBQ7*, which functioned as the control gene. Each experiment consisted of three technical replicates and three biological replicates.

## 5. Conclusions

This study represents an initial comprehensive investigation of the DUSP gene cluster in cotton. In four different varieties of cotton, we identified a total of 120 DUSP genes, with 32 found in *G. arboreum*, 42 in *G. barbadense*, 29 in *G. hirsutum*, and 17 in *G. raimondii*. These genes primarily originated from events such as whole-genome duplication (WGD) or fragment occurrences during evolutionary processes. We employed a phylogenetic tree analysis, along with an examination of gene structure and motifs, to categorize DUSP family genes and make predictions regarding cis-acting elements to gain insight into the biological collaboration pathways in which they are involved. At the same time, we determined the physical location, gene sequence, and structure of these DUSP genes on the chromosomes of the plants. The expression pattern of DUSP family genes was revealed using RNA-seq data, and the correlation between target genes and plants against *Verticillium wilt* was preliminarily verified using the VIGS technique. This study not only significantly enhanced our understanding of DUSP genes in upland cotton; it also provides reference for further research on bispecific phosphatases in other crops. Furthermore, the comprehensive examination of leaf tissue provides a basis for future investigations into the functional significance of DUSP family genes in cotton.

## Data Availability

The data presented in this study are available in the article and Supplementary Materials.

## Supplementary Materials

The following supporting information can be downloaded at https://www.mdpi.com/article/10.3390/ijms25084500/s1.
