# Supplementary material for: Revealing the Complete Bispecific Phosphatase Genes (DUSPs) across the Genome and Investigating the Expression Patterns of GH_A11G3500 Resistance against Verticillium wilt
Source: Int J Mol Sci. 2024 Apr 19;25(8):4500. doi: 10.3390/ijms25084500 (PMC11050305; doi:10.3390/ijms25084500)
Supplement: Supplementary file 1 [file ijms-25-04500-s001.zip › ijms-2911885-supplementary.pdf]

**Table S1.** Identification data pertaining to the members of the DUSP gene family in cotton.

| Gene ID | Gene Name | Genomic Location | Protein Length (bp) | Molecular Weight (Da) | Theoretical Isoelectric Point (pI) |
|---|---|---|---|---|---|
| Garb_01G029220. 1 | *GaDUSP01* | Chr01:122,867,721-122,874,311 | 562 | 62.09 | 5.76 |
| Garb_02G010450. 1 | *GaDUSP02* | Chr02:63,242,590-63,246,216 | 862 | 95.66 | 5.77 |
| Garb_03G003540. 1 | *GaDUSP03* | Chr03:2,609,193-2,613,999 | 584 | 64.43 | 5.82 |
| Garb_03G009860. 1 | *GaDUSP04* | Chr03:18,095,042-18,099,638 | 835 | 91.83 | 5.52 |
| Garb_04G002590. 1 | *GaDUSP05* | Chr04:3,628,079-3,634,581 | 380 | 43.10 | 6.77 |
| Garb_04G007170. 1 | *GaDUSP06* | Chr04:14,141,801-14,147,615 | 850 | 94.08 | 7.71 |
| Garb_05G027210. 1 | *GaDUSP08* | Chr05:29,484,863-29,488,012 | 289 | 33.11 | 8.88 |
| Garb_05G000860. 1 | *GaDUSP07* | Chr05:1,027,473-1,029,943 | 284 | 31.93 | 9.47 |
| Garb_06G004010. 1 | *GaDUSP09* | Chr06:5,412,199-5,415,391 | 268 | 29.96 | 9.24 |
| Garb_06G019920. 1 | *GaDUSP10* | Chr06:125,797,240-125,799,297 | 283 | 31.89 | 8.74 |
| Garb_06G019980. 1 | *GaDUSP11* | Chr06:125,853,301-125,860,991 | 286 | 33.41 | 9.00 |
| Garb_07G005680. 1 | *GaDUSP14* | Chr07:11,699,715-11,702,014 | 204 | 23.32 | 5.86 |
| Garb_07G001470. 1 | *GaDUSP12* | Chr07:1,835,544-1,840,133 | 564 | 63.15 | 8.86 |
| Garb_07G019800. 1 | *GaDUSP17* | Chr07:91,792,909-91,795,535 | 213 | 24.45 | 8.47 |
| Garb_07G009920. 1 | *GaDUSP15* | Chr07:51,693,786-51,697,905 | 214 | 24.51 | 6.70 |
| Garb_07G019730. 1 | *GaDUSP16* | Chr07:91,465,407-91,471,745 | 301 | 33.62 | 8.03 |
| Garb_07G005350. 1 | *GaDUSP13* | Chr07:9,913,113-9,916,885 | 268 | 29.79 | 7.56 |
| Garb_08G016040. 1 | *GaDUSP18* | Chr08:32,407,767-32,423,488 | 1211 | 136.82 | 6.16 |
| Garb_08G028990. 1 | *GaDUSP19* | Chr08:137,528,557-137,539,928 | 1253 | 140.99 | 7.17 |
| Garb_09G029940. 1 | *GaDUSP21* | Chr09:89,341,873-89,347,500 | 935 | 104.10 | 6.00 |
| Garb_09G024410. 1 | *GaDUSP20* | Chr09:84,684,536-84,688,128 | 219 | 24.97 | 6.45 |
| Garb_10G027850. 1 | *GaDUSP23* | Chr10:123,774,782-123,779,809 | 838 | 93.20 | 6.5 |
| Garb_10G001060. 1 | *GaDUSP22* | Chr10:824,268-826,857 | 201 | 22.81 | 6.44 |
| Garb_11G017040. 1 | *GaDUSP29* | Chr11:19,759,668-19,763,372 | 271 | 30.23 | 6.50 |
| Garb_11G005460. 1 | *GaDUSP25* | Chr11:4,564,433-4,570,302 | 647 | 71.29 | 6.09 |
| Garb_11G011380. 1 | *GaDUSP28* | Chr11:10,679,780-10,695,664 | 1255 | 141.35 | 6.14 |
| Garb_11G008400. 1 | *GaDUSP27* | Chr11:7,192,982-7,196,091 | 314 | 35.31 | 5.93 |
| Garb_11G007890. 1 | *GaDUSP26* | Chr11:6,751,666-6,754,764 | 448 | 51.75 | 8.53 |
| Garb_11G040720. 1 | *GaDUSP30* | Chr11:134,410,353-134,415,804 | 364 | 40.41 | 6.04 |
| Garb_11G002830. 1 | *GaDUSP24* | Chr11:2,388,661-2,391,293 | 333 | 37.68 | 6.26 |
| Garb_12G002410. 1 | *GaDUSP31* | Chr12:3,209,340-3,215,620 | 618 | 67.18 | 5.79 |
| Garb_13G021030. 1 | *GaDUSP32* | Chr13:115,824,908-115,827,338 | 201 | 23.25 | 7.56 |
| Gbar_A01G015500. 1 | *GbDUSP01* | A01:94,311,099-94,315,503 | 862 | 95.88 | 5.76 |
| Gbar_A03G014990. 1 | *GbDUSP02* | A03:88,370,923-88,375,352 | 835 | 91.86 | 5.52 |
| Gbar_A05G039650. 3 | *GbDUSP06* | A05:99,190,595-99,197,143 | 380 | 43.16 | 6.53 |
| Gbar_A05G027510. 1 | *GbDUSP04* | A05:30,378,789-30,382,046 | 257 | 29.23 | 8.46 |
| Gbar_A05G035700. 7 | *GbDUSP05* | A05:89,681,064-89,686,978 | 850 | 93.99 | 7.93 |
| Gbar_A05G002500. 2 | *GbDUSP03* | A05:2,294,698-2,297,685 | 290 | 32.66 | 9.39 |
| Gbar_A06G007170. 1 | *GbDUSP07* | A06:15,791,657-15,793,786 | 283 | 31.89 | 8.74 |
| Gbar_A07G023420. 1 | *GbDUSP10* | A07:91,038,480-91,043,185 | 572 | 63.91 | 8.84 |
| Gbar_A07G019960. 1 | *GbDUSP09* | A07:83,658,744-83,663,102 | 268 | 29.86 | 7.57 |
| Gbar_A07G009100. 1 | *GbDUSP08* | A07:14,009,753-14,018,900 | 265 | 29.72 | 6.99 |
| Gbar_A09G019830. 3 | *GbDUSP11* | A09:71,931,759-71,938,578 | 932 | 105.62 | 5.79 |
| Gbar_A09G021150. 1 | *GbDUSP13* | A09:73,182,949-73,186,649 | 219 | 25.00 | 6.45 |
| Gbar_A09G026570. 3 | *GbDUSP14* | A09:77,812,657-77,818,804 | 935 | 104.12 | 6.05 |
| Gbar_A09G021140. 14 | *GbDUSP12* | A09:73,173,571-73,176,090 | 206 | 23.55 | 9.17 |
| Gbar_A10G020920. 1 | *GbDUSP16* | A10:100,207,985-100,210,453 | 138 | 16.15 | 9.78 |
| Gbar_A10G022730. 1 | *GbDUSP17* | A10:104,555,887-104,558,718 | 843 | 93.55 | 6.63 |
| Gbar_A10G020810. 2 | *GbDUSP15* | A10:99,928,853-99,942,122 | 959 | 107.21 | 5.34 |
| Gbar_A11G007900. 1 | *GbDUSP19* | A11:6,895,513-6,898,680 | 314 | 35.30 | 5.93 |
| Gbar_A11G002370. 1 | *GbDUSP18* | A11:1,995,428-1,998,475 | 333 | 37.67 | 6.26 |
| Gbar_A11G015770. 1 | *GbDUSP20* | A11:18,139,879-18,144,005 | 271 | 30.21 | 6.65 |
| Gbar_A12G025220. 2 | *GbDUSP21* | A12:98,915,995-98,923,036 | 960 | 107.76 | 5.66 |
| Gbar_D01G016790. 2 | *GbDUSP22* | D01:50,560,215-50,564,663 | 863 | 95.65 | 5.79 |
| Gbar_D02G016880. 1 | *GbDUSP23* | D02:55,269,200-55,273,296 | 835 | 92.07 | 5.62 |
| Gbar_D04G006430. 1 | *GbDUSP25* | D04:10,241,534-10,245,034 | 851 | 94.23 | 7.74 |
| Gbar_D04G002260. 3 | *GbDUSP24* | D04:3,019,344-3,025,906 | 376 | 42.59 | 6.53 |

| | | | | | |
|---|---|---|---|---|---|
| Gbar_D05G028370. 1 | *GbDUSP28* | D05:27,630,811-27,634,460 | 289 | 33.02 | 8.99 |
| Gbar_D05G010380. 2 | *GbDUSP27* | D05:8,419,712-8,423,063 | 284 | 30.88 | 5.43 |
| Gbar_D05G002770. 1 | *GbDUSP26* | D05:2,414,582-2,417,320 | 284 | 32.12 | 9.41 |
| Gbar_D06G007410. 1 | *GbDUSP29* | D06:11,706,045-11,708,179 | 283 | 31.78 | 8.90 |
| Gbar_D07G024180. 1 | *GbDUSP32* | D07:54,743,829-54,748,506 | 573 | 64.16 | 8.89 |
| Gbar_D07G009500. 1 | *GbDUSP30* | D07:11,325,590-11,331,040 | 265 | 29.68 | 6.65 |
| Gbar_D07G020550. 1 | *GbDUSP31* | D07:48,257,475-48,261,900 | 264 | 29.35 | 6.65 |
| Gbar_D09G026140. 2 | *GbDUSP36* | D09.51,393,288-51,399,490 | 935 | 104.11 | 6.12 |
| Gbar_D09G020860. 2 | *GbDUSP34* | D09:47,194,153-47,198,205 | 196 | 22.48 | 8.97 |
| Gbar_D09G020870. 1 | *GbDUSP35* | D09:47,203,407-47,206,430 | 176 | 19.97 | 4.59 |
| Gbar_D09G019590. 2 | *GbDUSP33* | D09:45,998,962-46,005,703 | 932 | 105.49 | 6.03 |
| Gbar_D10G021090. 1 | *GbDUSP37* | D10:55,834,905-55,848,216 | 959 | 107.40 | 5.45 |
| Gbar_D10G023050. 1 | *GbDUSP38* | D10:59,464,545-59,467,373 | 842 | 93.72 | 6.23 |
| Gbar_D11G008330. 2 | *GbDUSP40* | D11:6,506,996-6,510,159 | 314 | 35.39 | 5.58 |
| Gbar_D11G002750. 1 | *GbDUSP39* | D11:2,153,917-2,156,826 | 333 | 37.65 | 6.26 |
| Gbar_D11G016570. 1 | *GbDUSP41* | D11:15,806,240-15,810,374 | 271 | 30.31 | 8.09 |
| Gbar_D12G025140. 2 | *GbDUSP42* | D12:55,920,499-55,930,034 | 960 | 107.51 | 5.71 |
| GH_A03G1540 | *GhDUSP01* | A03:94,693,102-94,696,192 | 835 | 91.85 | 5.52 |
| GH_A05G0273 | *GhDUSP02* | A05:2,635,062-2,636,743 | 284 | 31.92 | 9.39 |
| GH_A05G3715 | *GhDUSP03* | A05:97,483,554-97,487,059 | 850 | 93.99 | 8.21 |
| GH_A05G4143 | *GhDUSP04* | A05:107,427,955-107,434,224 | 378 | 42.87 | 6.77 |
| GH_A06G0743 | *GhDUSP05* | A06:16,229,295-16,230,945 | 283 | 31.89 | 8.74 |
| GH_A07G2141 | *GhDUSP07* | A07:87,676,806-87,680,577 | 268 | 29.91 | 7.56 |
| GH_A07G0942 | *GhDUSP06* | A07:13,592,945-13,598,509 | 265 | 29.63 | 6.08 |
| GH_A09G2171 | *GhDUSP08* | A09:78,560,777-78,564,044 | 219 | 24.94 | 6.71 |
| GH_A09G2699 | *GhDUSP09* | A09:83,078,997-83,084,392 | 935 | 104.14 | 6.05 |
| GH_A10G2332 | *GhDUSP10* | A10:109,975,880-109,978,711 | 843 | 93.56 | 6.50 |
| GH_A11G3500 | *GhDUSP14* | A11:118,306,098-118,308,968 | 364 | 40.41 | 5.68 |
| GH_A11G0784 | *GhDUSP12* | A11:6,964,216-6,966,230 | 314 | 35.30 | 5.93 |
| GH_A11G1592 | *GhDUSP13* | A11:18,498,426-18,501,845 | 271 | 30.21 | 6.65 |
| GH_A11G0262 | *GhDUSP11* | A11:2,292,765-2,294,462 | 333 | 37.68 | 6.26 |
| GH_D01G1827 | *GhDUSP15* | D01:52,261,842-52,265,494 | 863 | 95.65 | 5.84 |
| GH_D04G0235 | *GhDUSP16* | D04:3,217,493-3,223,761 | 374 | 42.36 | 6.53 |
| GH_D04G0631 | *GhDUSP17* | D04:10,520,729-10,524,229 | 851 | 94.23 | 7.74 |
| GH_D05G2823 | *GhDUSP19* | D05:27,117,549-27,120,710 | 289 | 33.02 | 8.99 |
| GH_D05G0277 | *GhDUSP18* | D05:2,362,176-2,363,822 | 284 | 32.06 | 9.33 |
| GH_D06G0719 | *GhDUSP21* | D06:11,938,249-11,939,899 | 283 | 31.81 | 8.53 |
| GH_D06G0537 | *GhDUSP20* | D06:7,531,025-7,532,938 | 637 | 74.62 | 9.03 |
| GH_D07G0943 | *GhDUSP22* | D07:10,878,251-10,882,610 | 265 | 29.69 | 6.70 |
| GH_D07G2079 | *GhDUSP23* | D07:50,441,944-50,445,755 | 264 | 29.32 | 7.03 |
| GH_D09G2619 | *GhDUSP24* | D09.51,895,766-51,901,170 | 935 | 104.11 | 6.12 |
| GH_D10G2426 | *GhDUSP25* | D10:61,625,657-61,628,491 | 844 | 93.88 | 6.14 |
| GH_D11G3506 | *GhDUSP29* | D11:68,214,494-68,217,381 | 364 | 40.36 | 5.79 |
| Grai_01G018320. 1 | *GrDUSP01* | Chr01:40,368,261-40,371,908 | 863 | 95.72 | 5.85 |
| Grai_03G019930. 1 | *GrDUSP02* | Chr03:50,769,381-50,773,843 | 832 | 91.53 | 5.44 |
| Grai_04G022070. 1 | *GrDUSP04* | Chr04:47,528,061-47,533,544 | 402 | 45.47 | 8.66 |
| Grai_04G017440. 1 | *GrDUSP03* | Chr04:40,613,521-40,619,334 | 851 | 94.20 | 7.18 |
| Grai_05G002900. 1 | *GrDUSP05* | Chr05:2,229,317-2,231,860 | 284 | 32.12 | 9.41 |
| Grai_05G029900. 1 | *GrDUSP06* | Chr05.25,831,231-25,834,375 | 289 | 32.98 | 8.88 |
| Grai_06G024140. 1 | *GrDUSP09* | Chr06:56,591,393-56,594,756 | 268 | 30.06 | 9.33 |
| Grai_06G024130. 1 | *GrDUSP08* | Chr06:56,567,355-56,571,062 | 268 | 29.99 | 9.24 |
| Grai_06G008210. 1 | *GrDUSP07* | Chr06:11,314,637-11,316,808 | 283 | 31.78 | 8.9 |
| Grai_07G005730. 1 | *GrDUSP10* | Chr07:8,073,067-8,077,391 | 264 | 29.35 | 7.03 |
| Grai_07G019420. 1 | *GrDUSP11* | Chr07:42,623,832-42,628,755 | 265 | 29.61 | 6.59 |
| Grai_09G029320. 1 | *GrDUSP12* | Chr09:49,833,934-49,840,220 | 876 | 97.60 | 6.07 |
| Grai_10G027350. 1 | *GrDUSP13* | Chr10:56,896,256-56,901,358 | 842 | 93.70 | 6.23 |
| Grai_11G002750. 1 | *GrDUSP14* | Chr11:2,633,331-2,638,880 | 364 | 40.32 | 5.68 |
| Grai_11G031300. 1 | *GrDUSP16* | Chr11:57,022,682-57,025,519 | 314 | 35.34 | 5.69 |
| Grai_11G023160. 1 | *GrDUSP15* | Chr11:48,405,951-48,409,723 | 271 | 30.25 | 7.58 |
| Grai_11G036870. 1 | *GrDUSP17* | Chr11:61,190,843-61,192,543 | 333 | 37.65 | 6.26 |
